# Detecting hierarchical levels of connectivity in a population of *Acacia tortilis* at the northern edge of the species’ global distribution: Combining classical population genetics and network analyses

**DOI:** 10.1371/journal.pone.0194901

**Published:** 2018-04-12

**Authors:** Yael S. Rodger, Gili Greenbaum, Micha Silver, Shirli Bar-David, Gidon Winters

**Affiliations:** 1 Mitrani Department of Desert Ecology, The Jacob Blaustein Institutes for Desert Research, Ben-Gurion University of the Negev, Midreshet Ben-Gurion, Israel; 2 The Dead Sea-Arava Science Center, Tamar Regional Council, Neve Zohar, Israel; 3 Department of Biology, Stanford University, Stanford, California, United States of America; 4 The Remote Sensing Laboratory, The Jacob Blaustein Institutes for Desert Research, Ben-Gurion University of the Negev, Midreshet Ben-Gurion, Israel; Universita degli Studi della Tuscia, ITALY

## Abstract

Genetic diversity and structure of populations at the edge of the species’ spatial distribution are important for potential adaptation to environmental changes and consequently, for the long-term survival of the species. Here, we combined classical population genetic methods with newly developed network analyses to gain complementary insights into the genetic structure and diversity of *Acacia tortilis*, a keystone desert tree, at the northern edge of its global distribution, where the population is under threat from climatic, ecological, and anthropogenic changes. We sampled *A*. *tortilis* from 14 sites along the Dead Sea region and the Arava Valley in Israel and in Jordan. In addition, we obtained samples from Egypt and Sudan, the hypothesized origin of the species. Samples from all sites were genotyped using six polymorphic microsatellite loci.Our results indicate a significant genetic structure in *A*. *tortilis* along the Arava Valley. This was detected at different hierarchical levels—from the basic unit of the subpopulation, corresponding to groups of trees within ephemeral rivers (wadis), to groups of subpopulations (*communities*) that are genetically more connected relative to others. The latter structure mostly corresponds to the partition of the major drainage basins in the area. Network analyses, combined with classical methods, allowed for the identification of key *A*. *tortilis* subpopulations in this region, characterized by their relatively high level of genetic diversity and centrality in maintaining gene flow in the population. Characterizing such key subpopulations may enable conservation managers to focus their efforts on certain subpopulations that might be particularly important for the population’s long-term persistence, thus contributing to species conservation within its peripheral range.

## Introduction

Identifying the spatial patterns of a species’ genetic diversity can inform conservation priorities, enabling management and conservation efforts to focus on particular populations [1,2]. Of particular interest are populations at the edge of a species’ spatial distribution, which studies indicate are important for a species’ long-term survival and evolution [3,4].

A growing number of studies across species’ geographical ranges have reported high levels of genetic variability in peripheral areas [5–7]. They have also reported the presence of unique genotypes that are potentially important for adaptation to local or new conditions at the species’ periphery [8,9], and a relatively high persistence of populations at the periphery following range contraction [10]. Considering the potential effect of climate change on species distribution, reduced genetic variability at a species’ edge may restrict possible range expansion and potential for genetic adaptation [11]. These concerns call for the protection of peripheral populations [6,8,10].

Revealing the genetic diversity of peripheral populations, in particular of species that are of conservation concern, may provide an important basis for protection and management strategies. Specifically, understanding the genetic structure of edge populations, at different hierarchical levels (individual-subpopulation and subpopulation-population levels), and characterizing important subpopulations that are critical for maintaining gene flow and sustaining genetic diversity can contribute to the allocation of conservation and management resources to specific subpopulations within peripheral regions.

Network theory (also known as graph theory) approaches have been increasingly applied in conservation biology and population genetics [12,13]. A network is made up of discrete elements, *nodes*, which are connected by links or edges. In a population-genetic context, a node can represent either an individual or a group of individuals defined by habitat patches, sampling sites, or subpopulations; edges represent genetic similarity among individuals or genetic connectivity between patches/sites, depending on their role in the network. Network methodologies have been applied in identifying and analyzing population structure [13,14], as well as in identifying central subpopulations [12,15,16].

As in classical population structure inference methods (*Fst*, AMOVA), network methods have been used for inferring population genetic structure at different hierarchical levels. Specifically, network methods have been developed for analyzing structure at the individual-subpopulation level (i.e., identifying subpopulations; [14]), and for identifying genetic interactions between subpopulations at the subpopulation-population level [15]. However, unlike many classical population genetic methods, network theory methods are mostly free of *a priori* assumptions and account simultaneously for genetic relationships between all elements rather than relying on pairwise comparisons [14,16]. One network methodology that has proven useful for such hierarchical analyses is the detection of *communities*—densely connected groups of nodes [13]. Community detection has been used to identify subpopulations by detecting densely connected groups of individuals [14] and to assess population structure at the higher hierarchical level by detecting groups of subpopulations within a population [6,17,18].

Identifying subpopulations that have a key role in genetic processes is a major challenge in conservation genetics. Genetic diversity is a key aspect of a subpopulation’s importance, as subpopulations with higher genetic diversity may contribute more to the diversity of the entire population. Another aspect is their significance for gene flow in the entire system; however, such gene flow dynamics may be complex and difficult to derive from pairwise comparisons of subpopulations. A rich array of centrality measures has been developed in network theory, intended to measure centrality for different functions and processes in networks [19]. The use of the *random walk betweenness centrality* measure (*RWB*, [20]) is a measure that captures the undirected and stochastic nature of gene flow [13]. Therefore, it has been suggested as an appropriate centrality measure for modeling gene flow within a population as a whole and for highlighting central subpopulations.

**Fig 1:**
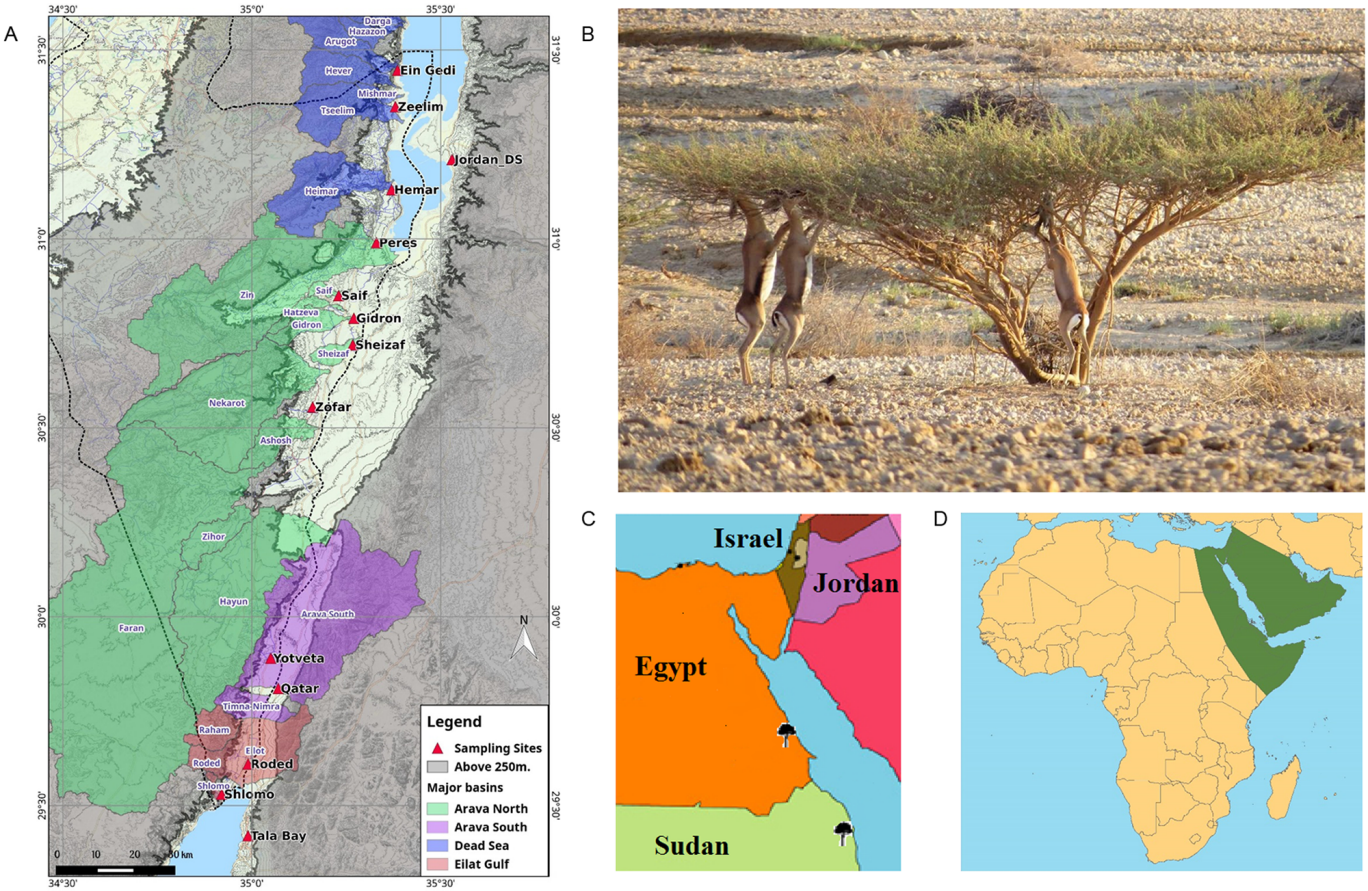
Study site and *A*. *tortilis* distributions. **a**: map of subpopulation sampling site distribution in Israel and Jordan. Also shown are the major drainage basins across the study area. **b**: acacia gazelles (*Gazella gazella acaciae* = *G*.*g*. *cora*) feeding on the foliage of *Acacia tortilis* at Yotveta Nature Reserve, Israel. Photo: Benny Shalmon. **c**: the location of the two sites from the species’ central distribution in Sudan and Egypt (black trees; map adapted from www.bjdesign.com). **d**: the approximate distribution of *A*. *tortilis*. This map is similar but not identical to a map published by FAO (http://www.fao.org/docrep/006/Q2934E/Q2934E05.htm), and is therefore for illustrative purposes only.

The Dead Sea region and the Arava Valley of Israel and Jordan (Fig 1a) hosts a population of *Acacia tortilis* (Forskk.) Hayne trees (Fig 1b; also known as *Vachellia tortilis*, [21]), growing at the northern edge of their global distribution (Fig 1d). These thorny desert trees grow in this hot, hyper-arid desert and are thought to be of Sudanese origin [22]. Considered a keystone species in this region [23], the trees provide shelter and vital forage for many desert animals and increase plant species diversity under their canopies by improving soil conditions [24–26](Fig 1d). However, this population is under threat from climatic, ecological, and anthropogenic changes [27]. An analysis of long-term rainfall data in the region showed a decline in precipitation levels and patterns within the Arava [27]. Annual precipitation in this arid region has always been low (2550 mm year^-1^ [28]), but a recent study [29] has shown that precipitation levels during the past 15 years have declined even further (25–30 mm year^-1^), as well as indicating to changes in the timing and duration of flood events, which are critical for acacia trees [29]. Studies have recorded increased rates of pod infestation by bruchid beetles [30], as well as low recruitment of seedlings [31]. Reduction in large mammalian herbivores, crucial for seed dispersal and germination success, could be another factor in the population decline [32,33], as well as anthropogenic pressures such as road-building [34].

In this study, we applied a combination of classical population genetic methods and network analysis procedures in order to understand the population structure of *A*. *tortillis* in the Arava Valley of Israel and Jordan, aiming to guide future management efforts in the region. We examined connectivity within the population at different hierarchical levels (subpopulations and *communities* of subpopulations) and identified key subpopulations that may be particularly important for maintaining gene flow and diversity at the northern edge of *A*. *tortilis* global distribution.

## Materials and methods

### Study area and population sampling

This study was conducted along the Dead Sea region, the Arava Valley and the Gulf of Eilat/Aqaba in Israel and Jordan (Fig 1a). The elevation of the area ranges from 230 m above sea level to 419 m below sea level. Summer maximum air temperatures reach 35–40°C, dropping to ~10°C in the coldest months [28]. Average annual precipitation is less than 50 mm, with large year-to-year variations [29]. Most of the vegetation in the region is confined within wadis (ephemeral river beds; [35]), where the main water supply comes from underground aquifers [36] and winter flash floods [37].

Fourteen sites within wadis were sampled in Israel and Jordan, spanning approximately 228 km throughout the *Acacia tortilis*’s northernmost distribution area (Fig 1d). Each sampling site included a group of *A*. *tortilis* trees (Fig 1b) growing within a wadi. Following previous research on *A*. *raddiana* in Israel [38], we predicted that the sampling sites would represent different *A*. *tortilis* subpopulations that are effectively isolated from each other. Most of the sampled wadis can be assigned to one of four major drainage catchment areas: the Gulf of Eilat/Aqaba, Arava South, Arava North and the Dead Sea (Fig 1a).

We collected 5–10 leaves from 15–24 *A*. *tortilis* trees within each sampling site, for a total of 318 trees (Table 1). Leaf samples were dried and stored with silica beads. In addition, leaf samples from two other sites in Egypt and Sudan, the hypothesized origin of the species ([22,38]; Fig 1c and 1d), were provided by Knut Krzywinski (Bergen University, Norway). These samples provided us with information regarding the genetics of the species at the commencement site of its global distribution (Fig 1c and 1d).

**Table 1:** Study site locations and associated genetic diversity and centrality measures.

Location	Country	Lat (°N)	Long (°E)	Elevation (m)	# Samples (n)	N	N_P_	AR	H_O_	H_E_	F	RWB
Ein Gedi	Israel	31.45	35.39	-371	21	6.83 *(0*.*54)*	0.17	5.26 *(0*.*52)*	0.73 *(0*.*05)*	0.71 *(0*.*03)*	-0.02 *(0*.*05)*	0.26
Zeelim	Israel	31.35	35.38	-343	24	7.00 *(0*.*52)*	0.17	5.33 *(0*.*54)*	0.80 *(0*.*03)*	0.72*(0*.*02)*	-0.12 *(0*.*03)*	0.26
Jordan_DS	Jordan	31.21	35.53	-362	24	9.33 *(1*.*05)*	0	6.67 *(1*.*13)*	0.79 *(0*.*04)*	0.78 *(0*.*02)*	-0.01 *(0*.*05)*	0.30
Hemar	Israel	31.13	35.37	-344	20	7.33 *(0*.*59)*	0.17	6.22 *(1*.*20)*	0.78 *(0*.*04)*	0.76 *(0*.*03)*	-0.02 *(0*.*04)*	0.31
Peres	Israel	30.99	35.33	-301	15	5.83 *(0*.*48)*	0	5.26 *(0*.*93)*	0.70 *(0*.*05)*	0.71 *(0*.*03)*	0.02 *(0*.*07)*	0.34
Saif	Israel	30.85	35.23	-88	17	7.83 *(0*.*60)*	0	6.35 *(0*.*85)*	0.83 *(0*.*02)*	0.76 *(0*.*02)*	-0.10 *(0*.*02)*	0.26
Gidron	Israel	30.79	35.27	-148	20	7.67 *(0*.*76)*	0	6.04 *(0*.*81)*	0.74 *(0*.*07)*	0.76 *(0*.*02)*	0.03 *(0*.*07)*	0.32
Sheizaf	Israel	30.72	35.27	-137	24	8.33 *(0*.*84)*	0	6.12 *(0*.*98)*	0.78 *(0*.*05)*	0.77 *(0*.*02)*	-0.01 *(0*.*05)*	0.28
Zofar	Israel	30.56	35.16	24	21	7.17 *(0*.*40)*	0.33	5.61 *(0*.*66)*	0.79 *(0*.*03)*	0.73 *(0*.*03)*	-0.08 *(0*.*02)*	0.36
Yotveta	Israel	29.89	35.05	71	23	8.50 *(1*.*03)*	0.67	6.49 *(1*.*36)*	0.81 *(0*.*05)*	0.77 *(0*.*03)*	-0.04 *(0*.*03)*	0.29
Qatar	Jordan	29.81	35.07	72	20	9.17 *(0*.*87)*	0.17	6.87 *(0*.*88)*	0.73 *(0*.*03)*	0.76 *(0*.*02)*	0.04 *(0*.*05)*	0.36
Roded	Israel	29.61	34.99	27	21	9.00 *(1*.*13)*	0.50	6.73 *(1*.*23)*	0.73 *(0*.*03)*	0.79 *(0*.*01)*	0.07 *(0*.*04)*	0.27
Shlomo	Israel	29.53	34.92	171	18	6.67 *(0*.*56)*	0.17	5.35 *(0*.*85)*	0.63 *(0*.*04)*	0.64 *(0*.*05)*	0.01 *(0*.*03)*	0.25
Tala Bay	Jordan	29.42	34.99	70	24	9.50 *(0*.*81)*	0.33	6.81 *(1*.*12)*	0.76 *(0*.*03)*	0.76 *(0*.*02)*	0.00 *(0*.*05)*	0.25
**Mean**						**7.87 *(1*.*09)***	**0.19 *(0*.*20)***	**6.08 *(0*.*59)***	**0.76 *(0*.*05)***	**0.74 *(0*.*04)***	**-0.08 *(0*.*01)***	**0.29**
Egypt	Egypt	24.40	35.10	156	14	6.33 *(1*.*15)*	1.83	5.62 *(1*.*86)*	0.53 *(0*.*06)*	0.71 *(0*.*03)*	0.26 *(0*.*06)*	
Sudan	Sudan	18.99	30.86	253	12	9.00 *(0*.*63)*	1.5	7.98 *(0*.*05)*	0.72 *(0*.*05)*	0.81 *(0*.*02)*	0.11 *(0*.*04)*	

Shown are locations (name, country, latitude, longitude and elevation), number of samples from each location (n; in parentheses), number of alleles per sample (N), unique or private alleles per locus (N_p_), allelic richness (AR), observed (H_O_) and expected (H_e_) heterozygosity, Fixation Index or inbreeding coefficient (F) and the random walk betweenness centrality measure (RWB).

### DNA extraction, amplification, and genotyping

Total genomic DNA was extracted from each sample, using 20 mg of silica gel-dried leaf material, with the DNeasy Plant Mini Kit (QIAGEN Inc., Valencia, USA). DNA samples were amplified using polymerase chain reactions (PCR) with eight fluorescent microsatellite primers developed for *A*. *tortilis* according to the PCR procedure detailed in Winters et al. [39]. PCR products were sent for genotyping with an ABI PRISM 3730xl DNA Analyzer (Applied Biosystems, Life Technologies) at the Center for Genomic Technologies at the Hebrew University of Jerusalem (http://www.bio.huji.ac.il/). Fragments were scored using Peak Scanner v1.0 software (Applied Biosystems, Life Technologies) to obtain a DNA profile of each individual tree. To account for error rates in the genotyping data set, a random selection of ~15% of all samples (*n* = 48) was independently re-genotyped [40]. Estimated error rates of < 5% were deemed acceptable.

### Measurements of genetic diversity

Tests for linkage disequilibrium among all loci pairs in all sampling sites and tests for significant deviations from the Hardy-Weinberg equilibrium (HWE) at each locus in each sampling site were performed using Genepop [41]. Possible genotyping errors (e.g., null alleles and allele dropout) were checked using MICRO-CHECKER v2.2.3 [42].

To determine the genetic diversity levels in the population, allele frequencies, the average number of unique alleles, and the observed (H_O_) and expected (H_E_) heterozygosity were calculated for each sampling site in GenAlEx v6.501 [43]. Allelic richness was estimated for each locus and each sampling site in FSTAT v2.9.3 [44] using the rarefaction method, thus accounting for sample size differences.

### Detection of population genetic structure at different hierarchical levels

We used a combination of classical population genetics methods and network theory methods to infer population structure at different hierarchical levels:

### Individual–subpopulation level: Identification of subpopulations

We first tested our prediction that sampling sites represent different subpopulations, i.e., that wadis are the basic units of the population structure, using a network analysis and the NetStruct ([14] see also S1 Text). This method clusters individuals based only on genetic information and is a model-free approach without *a priori* assumptions. It constructs networks of individuals connected by edges characterized by their genetic similarity. Dense substructures, termed *communities*, are then detected in the networks. Edges are removed below a threshold of genetic similarity in order to account for different hierarchical levels of population structure.

Our null hypothesis was that sampling sites are not associated with genetic clusters of individuals. We used the FastGreedy community-detection algorithm [45] to test the null hypothesis by systematically removing low-value edges from the network, below fixed edge-removal thresholds (see S3 Fig for details). The hypothesis test was done using Fisher’s exact test for each edge-removal threshold. Rejection of the null hypothesis suggests that sampling sites are associated with population structure. We, conservatively, considered the null hypothesis rejected if it was rejected for all edge-removal thresholds tested (S3 Fig).

### Subpopulation–population level: Identification of genetic interactions between subpopulations

To examine genetic relationships between subpopulations (defined as sampling sites, following the analysis above), overall and pairwise estimates of genetic divergence (*Fst*) between subpopulations were calculated [46], with significance values generated by 999 permutations, using GenAlEx v6.501 [43]. An analysis of molecular variance (AMOVA) was also performed in GenAlEx with 999 permutations to estimate the levels of molecular variation within and between subpopulations.

It has been argued that estimating *Fst* from microsatellites can be problematic as the computation is affected by levels of genetic variability such as that the theoretical upper limit of an *Fst* estimate is not, in fact, 1. Hedrick [47] suggested that when various variable loci are used in calculations of *Fst*, the upper bound of *Fst* becomes deflated, and concurrently, Charlesworth [48] suggested that *Fst* can be inflated when diversity is low. Jakobsson et al. [49] showed that *Fst* can be strictly bounded by the functions of the frequency of the most common allele (*M*) and the total homozygosity of the total population (*H*_*T*_). Hence, the upper bounds of *Fst* were calculated for each locus across the population and then averaged over all loci [50].

Isolation-by-distance between subpopulations was tested using the Mantel permutation method implemented in GenAlEx v6.501 [43]. Two different matrices representing genetic distances were tested against a matrix of geographic distances between subpopulations: 1) a traditional matrix of linearized pairwise *Fst* values [51]; and 2) the “conditional genetic distance” (*cGD*) obtained using a network analysis [15]. A population graph of *A*. *tortilis* was constructed using POPGRAPH [52] (detailed below). The *cGD* was extracted between each pair of subpopulations as estimated by the shortest path connecting them within the population graph. STRUCTURE v2.3.4 [53] was used to detect population structure and to evaluate the most likely K (number of clusters). Using an admixture model with *a priori* information about sampling location [54], 15 independent runs were conducted for each value of K (ranging from 1–14, the number of subpopulations sampled). Each run consisted of a 50,000 burn-in period followed by 50,000 Markov Chain Monte Carlo (MCMC) estimations. The most likely K was estimated using the ΔK method following Evanno et al. [55], implemented in CLUMPAK [56].

### Community detection

Network analysis was used to identify “groups of subpopulations” (*communities* in a subpopulation-population network). We constructed a population graph for *A*. *tortilis* using POPGRAPH [15], whereby each subpopulation (sampling site) was represented by a node, and edges connected nodes with non-conditionally independent allele frequencies. The weight of each edge was defined as the within-site genetic variation, following Dyer and Nason [15]. POPGRAPH does not apply any *a priori* assumptions about the geographic arrangement of the subpopulations. We used a spectral decomposition method [18], implemented in igraph [57], to detect groups of subpopulations that were genetically densely connected within the network.

### Identifying important subpopulations

Network methods were used to identify key (“hotspot”) subpopulations of particular importance for maintaining gene flow and genetic diversity in the *A*. *tortilis* metapopulation. In the population graph described above, each node (sampling site) was characterized by:

1. The level of within-site genetic diversity as determined by heterozygosity and allelic richness.
2. The degree of centrality for gene flow in the network, determined by the RWB measure [20].

Various centrality measures have been developed to enable the identification of central nodes in networks [13,19]. Since we aimed to understand gene flow through nodes, the most appropriate centrality measures were flow measures, with the most commonly used measure being betweenness centrality [15,58,59]. *Betweenness* measures the amount of flow through nodes assuming that flow only occurs along the shortest paths, where a shortest path is a sequence of edges that connects nodes in a graph and minimizes the weight of the edges from which it is composed. However, it has been noted that since gene flow in natural populations does not act in such a confined and deterministic way, a more appropriate measure for modeling gene flow is one that does not limit flow only to the shortest paths but rather considers random walks, such as RWB [13]. Other measures that are non-deterministic and that could be considered are random walk closeness and eigenvector centrality [60]. However RWB is expected to score highly nodes that bridge different regions of the network, which would highlight subpopulations that are crucial for allowing gene flow to adequately reach different regions. We therefore chose RWB as the relevant centrality measure for our system, noting that this centrality measure also allows edge weights to be incorporated in the centrality assessment.

## Results

No significant linkage disequilibrium was detected for any pair of loci. Two of the eight loci, L3 and L9, deviated significantly from HWE in all subpopulations. The MICRO-CHECKER analysis suggested that these loci might have been affected by null alleles, and consequently, they were removed; in total, six loci were included in the analyses presented below.

### Levels of genetic diversity

The mean number of alleles per locus was 18.83, ranging from 15 to 26 (S1 Table). An average of 0.19 unique alleles per locus was found, and mean allelic richness (based on a minimum sampling size of nine individuals) was 6.08 ± 0.59 and ranged from 5.26 (Ein Gedi) to 6.87 (Qatar) (Table 1). High levels of genetic diversity were revealed across the population, particularly in the Jordan subpopulations (Jordan_DS, Qatar, Tala Bay). The central Arava subpopulations appeared to have fewer unique alleles (mean = 0.07) than subpopulations in the extreme north (mean = 0.13) and south of the distribution (mean = 0.37). Most of the inbreeding coefficient values (Fixation Index, F, Table 1) indicated random mating as they were close to zero [43]. Levels of genetic diversity within the population sampled in Egypt were low, with all measures below the mean for the Israel/Jordan population. However, in the Sudan population, levels of genetic diversity were relatively high (Table 1).

### Population genetic structure

#### Individual–subpopulation level

Analysis at the individual-subpopulation level aimed to examine whether sampling sites could be regarded as basic units of population structure, was performed by testing the null hypothesis that population structure is independent of assignment of individuals to sampling sites. The analysis examined different genetic similarity networks with different edge-removal thresholds, and in each, the null hypothesis was rejected (p < 0.05 for all thresholds and p < 0.0001 for most thresholds; S4 Table. S3 Fig shows an example of community partition for a specific edge-removal threshold). The detected clusters did not fully correspond to sampling sites, as expected, and differed by different edge-removal thresholds. The rejection of the null hypothesis indicates an association between sampling sites and population structure. Since sampling sites were the smallest spatial unit measured, we regarded sampling sites as the basic units of population structure, subpopulations, for further analysis at the subpopulation-population level.

#### Subpopulation–population level

Most pairwise *Fst* values indicated significant genetic differences between *A*. *tortilis* subpopulations (85% of pairwise comparisons, p < 0.05; S3 Table), supporting the proposition that sampling sites (wadis) constitute the basic units of the population structure. The AMOVA results showed further evidence for significant differentiation in the *A*. *tortilis* population in Israel and Jordan, with an overall population *Fst* of 0.036 (p < 0.001). The calculated upper limit (*F*) for *Fst* revealed that *Fst* was bound by the level of homozygosity and not the frequency of the most common allele, with results for the upper limits of 0.341 and 0.429, respectively. Hence, the *Fst* obtained for this population (0.036) was evaluated within the range of 0 to 0.341 instead of 0 to 1.

Genetic differentiation between subpopulations, as measured by *Fst* values, correlated positively with the geographic distance between them (Mantel test, r = 0.31, p = 0.01; S2A Fig). The same pattern, though significantly higher, was obtained when testing for isolation-by-distance using the *cGD* measure (r = 0.56, p < 0.01, S2B Fig).

**Fig 2:**
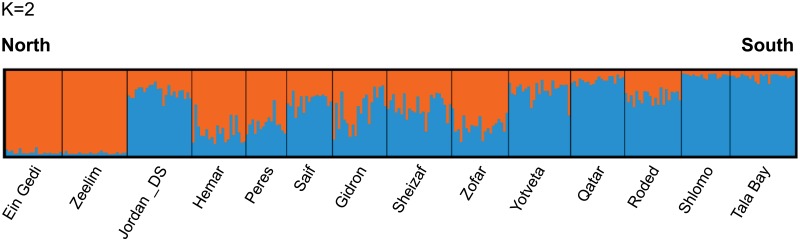
Subpopulation clustering results from STRUCTURE for K = 2. Each individual is denoted by a thin vertical line, partitioned into K-colored segments that represent the individual’s probability of membership fraction in K clusters. Black lines separate individuals of different subpopulations.

The results obtained from STRUCTURE confirmed a pronounced population genetic structure. The Evanno method suggested that the most likely number of clusters is K = 2 (S1 Fig). At K = 2, STRUCTURE identified a geographic gradient of differentiation from north to south (Fig 2).

#### Community detection

**Fig 3:**
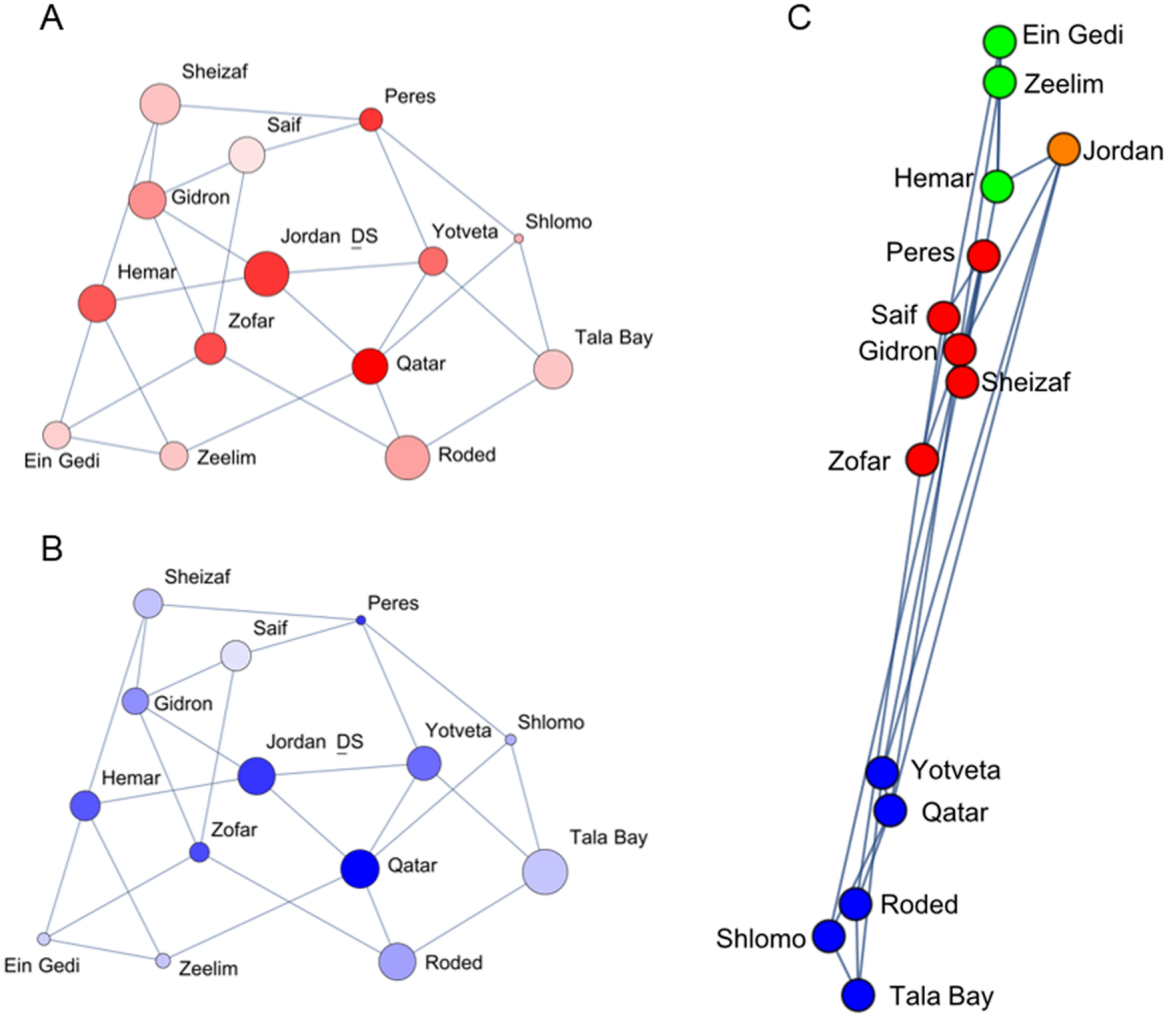
The genetic networks obtained from POPGRAPH. Shown are networks for all subpopulations in Israel and Jordan with node size representing within-site genetic diversity as **a**. observed heterozygosity and **b**. allelic richness. Node color shade represents the degree of RWB centrality, with darker color indicating a higher degree. **c**. Community structure in the genetic network of the *A*. *tortilis* tree population in Israel and Jordan, as determined by spectral-decomposition community detection [18]. Different node colors indicate membership in different communities detected by the algorithm. Node size represents genetic diversity as measured by the observed heterozygosity.

The genetic network obtained from POPGRAPH is shown in Fig 3. The community-detection algorithm revealed a further substructuring of the population: a partition into four communities (Fig 3c). This partition corresponded with the geographic locations of the subpopulations along the species’ north-south distribution and fit the division of the main drainage basins in the area (the Gulf of Eilat/Aqaba, the Arava South, the Arava North and the Dead Sea basins; Fig 1a).

#### Subpopulation centrality

In the genetic network (Fig 3), each subpopulation is presented by its genetic diversity (observed heterozygosity and allelic richness) and the degree of gene flow (RWB). The Jordan subpopulations, Qatar and Jordan_DS, appear to be the most central sites for gene flow in the entire network, having both high RWB and high genetic diversity (Fig 3a and 3b). Considering both the community-detection (Fig 3c) and centrality analyses, each of the four communities included a central subpopulation in terms of gene flow: Qatar in the southern community, and Peres, Hemar, and Jordan_DS in the northern communities. However, subpopulations with high genetic diversity were not necessarily associated with high RWB, and vice versa. For example, the Peres subpopulation had relatively low genetic diversity but a high degree of RWB, and Sheizaf, in the same community (Fig 3c), had high genetic diversity but relatively low RWB. These findings highlight the complexity in defining key subpopulations for conservation purposes.

## Discussion

The combination of classical population genetic procedures with network analysis procedures used here enabled us to gain complementary insights into the population genetic structure and diversity of *A*. *tortillis* at the northern edge of its world distribution. The peripheral population of *A*. *tortillis* is significantly structured at different levels, from the basic unit, the subpopulation (groups of trees within wadis), to groups of subpopulations that are genetically connected, located along the species’ geographic distribution. Subpopulations of particular genetic importance were identified in each of the communities. These key subpopulations may play an important role in maintaining gene flow and diversity in the population.

### Patterns of genetic diversity

High levels of genetic diversity were found across the population of *A*. *tortilis* in Israel and Jordan, contrary to what would be expected from apparently isolated groups of trees within wadis, growing at the northern edge of the population’s world distribution. The estimates of the heterozygosity values were much higher than those found for related species such as *A*. *senegal* [61], and *A*. *raddiana* ([62], using allozyme loci). These values and the average Fixation Index indicated that inbreeding was unlikely to be a concern in the population (Table 1). The subpopulations of Saif and Zeelim showed a slightly negative value, which may indicate disassortative mating at these sites, i.e., mating between plants with dissimilar genotypes may have occurred more frequently than under random mating. The populations sampled in Sudan and Egypt, however, showed positive values for the Fixation Index, suggesting that inbreeding may be occurring in these populations.

A study on *A*. *raddiana* in Israel also demonstrated high genetic diversity [38], and a few other population genetic studies of long-lived desert perennials showed this pattern as well [63–65]. Strong relationships between levels of genetic variability and the degree of environmental heterogeneity and stress have been documented [47,66]. The Dead Sea region and Arava Valley are characterized as hyper-arid and heterogeneous environments [29], which may have contributed to the maintenance of high levels of genetic diversity across the population of *A*. *tortilis*. In order to quantify relationships between genetic diversity and environmental variables, further studies should be performed using techniques such as genome-wide SNP genotyping, transcriptome profiling [67] and genotype x environment common stress garden experiments [68].

The relatively high levels of genetic diversity in the Israel and Jordan subpopulations highlight the need to protect *A*. *tortilis* at the northern edge of its global distribution. At the local scale, the rarity of unique alleles in the central subpopulations in the Arava (e.g., Peres, Saif, Gidron, Table 1) may indicate that there was, at least historically, gene flow between these central subpopulations, as well as between them and the northern and southern subpopulations. The potential connectivity between subpopulations should be further explored and considered in conservation and management strategies.

### Population genetic structure at different hierarchical levels revealed by the integration between classical and network methods

A significant genetic structure was detected in the *A*. *tortilis* population in Israel and Jordan, over a relatively small geographic range. The basic level of differentiation was predicted *a priori* to be the groups of trees sampled in distinct wadis. The network method, Netstruct, and the pair-wise *Fst* analysis (between wadis) supported this basic level of differentiation (subpopulations). Additionally, evidence was provided for a north-south gradient of differentiation. Firstly, STRUCTURE identified two clusters: the northern subpopulations were strongly associated with the first cluster and the southern subpopulations with the second cluster, while the central subpopulations had intermediate associations with both clusters. These results seem most consistent with the stepping-stone model of gene flow [69]. Secondly, significant isolation-by-distance (IBD) was found between subpopulations, using both conditional genetic distance (*cGD*) and linearized *Fst*. Results showed that the network-based measure, *cGD*, provided stronger evidence for IBD than the traditional *Fst* measure, as suggested by [52,70], since rather than calculating pairwise estimates of differentiation between subpopulations, *cGD* is based on a simultaneous analysis of the entire data set [52].

The isolation-by-distance model can explain a significant portion of the variation between subpopulations along the north-south gradient. However, the network analysis and the community detection algorithm revealed a further clustering of subpopulations into "communities of subpopulations" (Fig 3c). This substructuring indicates that there is additional genetic variation between subpopulations that cannot be explained solely by geographic distance. The general connection between the partitioning into four *A*. *tortilis* communities and the division into the four main drainage basins in the area (Fig 1a) provides a framework for further investigation into the connectivity between subpopulations and the centrality of subpopulations. Further studies should also examine the role of floods, pollination, animal movement and zoochory in gene flow within the acacia population [33,71].

The ability of network analysis to analyze population connectivity has not been fully explored [72] and has relevant applications for landscape genetics [58]. By visualizing genetic diversity and RWB centrality on the population graph, we identified key subpopulations with importance for diversity and gene flow in the population, which could allow management efforts to prioritize certain vital subpopulations to best conserve the peripheral *A*. *tortilis* population. In terms of genetic diversity, the highly diverse Jordan subpopulations, as well as Roded, Sheizaf, Gidron, and Saif, may serve as important “hot spots” for maintaining genetic diversity in the system [73,74]. Additionally, they may be considered as sources for future translocation efforts to increase the genetic diversity of other subpopulations.

For gene flow, the Qatar and Jordan_DS subpopulations in Jordan and Peres in Israel were identified as highly important. The Peres subpopulation, however, has comparatively low genetic diversity, perhaps as a result of a historical bottleneck experienced by the subpopulation. RWB quantifies the centrality of the node for gene flow in the entire system, and not the actual amount of gene flow experienced by the node. It may be possible to have either two peripheral nodes with extensive gene flow between them but not with other nodes (i.e., high gene flow, low RWB) or a single node that connects two regions but with relatively little gene flow between them (i.e., little gene flow, high RWB). Thus, RWB may be a more appropriate measure than other connectivity measures for identifying conservation priorities, in order to maintain gene flow throughout a population. When analyzing population genetic data for the purpose of prioritizing management efforts, one should consider the complexity in defining key subpopulations. As highlighted in this study, subpopulations with high genetic diversity are not necessarily associated with high gene flow with other subpopulations.

## Conclusions

In this study we revealed the population structure of *A*. *tortilis* at the edge of its global distribution, at different hierarchical levels: from the basic unit of the subpopulation, corresponding to groups of trees within ephemeral rivers, to groups of genetically connected subpopulations (communities). These results may help in making conservation decisions aimed at maintaining gene flow throughout the population. The use of a combination of classical and novel network theory applications provided complementary insights into understanding patterns of genetic diversity and levels of connectivity within a species. Specifically, these methods, combined, allowed for the identification of key *A*. *tortilis* subpopulations, which have relatively high level of genetic diversity and are central in maintaining gene flow in the entire population. Identifying key subpopulations may enable conservation managers to focus their efforts on specific subpopulations that are potentially crucial for the species conservation within its peripheral range.

## Supporting information

S1 Fig Results of the STRUCTURE analysis showing values of best K according to ΔK by Evanno et al. (2005) to identify optimal K. (DOCX)

S2 Fig Mantel tests for (A) pairwise linearized *Fst* and (B) conditional genetic distance (*cGD*) against geographic distance. (DOCX)

S3 Fig An example of NetStruct output for a specific edge-removal threshold (threshold = 0, i.e., no edges removed from the network). Nodes represent individual trees, with positions approximately corresponding to sampling sites; edges, in gray, represent genetic similarity. Node colors indicate the detected communities using NetStruct, which are determined independently of sampling site and without any *a priori* assumptions or underlying model. The distribution of communities in the population is not independent of sampling sites, with a rejection of the null hypothesis at p-value < 0.0001 (see S4 Table).
(DOCX)

S1 Table Allele frequencies and sample size for all subpopulations of *Acacia tortilis*. (DOCX)

S2 Table Primer name, locus, sequence, allele size range and fluorescent dye (forward primers were dye-labeled) for amplification of eight microsatellite loci in *Acacia tortilis*. (DOCX)

S3 Table Pairwise genetic distance (*Fst*) between subpopulations of the *A*. *tortilis* population in Israel and Jordan. *Fst* values are below the diagonal; p-values based on 999 permutations are shown above the diagonal.
(DOCX)

S4 Table Test results of the hypothesis that sampling sites correspond with population structure using NetStruct. The table lists, for all tested edge-removal thresholds, the number of communities detected using the FastGreedy algorithm in Netstruct, the number of connected nodes in the network after edge removal, and the p-value of the Fisher’s exact test of rejecting the null hypothesis that detected communities that are not dependent on sampling sites. All p-values are significant (p < 0.05), regardless of the edge-removal threshold used.
(DOCX)

S1 Text NetStruct analysis. (DOCX)
