# Supplementary figures and images for: Detecting hierarchical levels of connectivity in a population of Acacia tortilis at the northern edge of the species’ global distribution: Combining classical population genetics and network analyses
Source: PLoS One. 2018 Apr 12;13(4):e0194901. doi: 10.1371/journal.pone.0194901 (PMC5896914; doi:10.1371/journal.pone.0194901)

**S1 Fig**


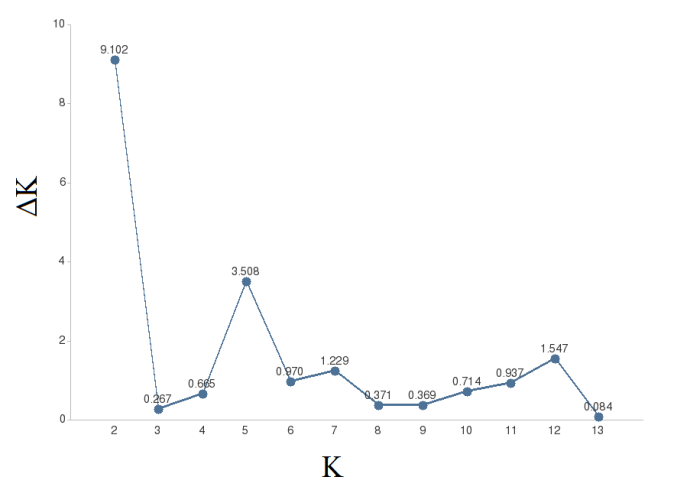

Supplement: S1 Fig — (DOCX) [file pone.0194901.s001.docx]

**S2 Fig**


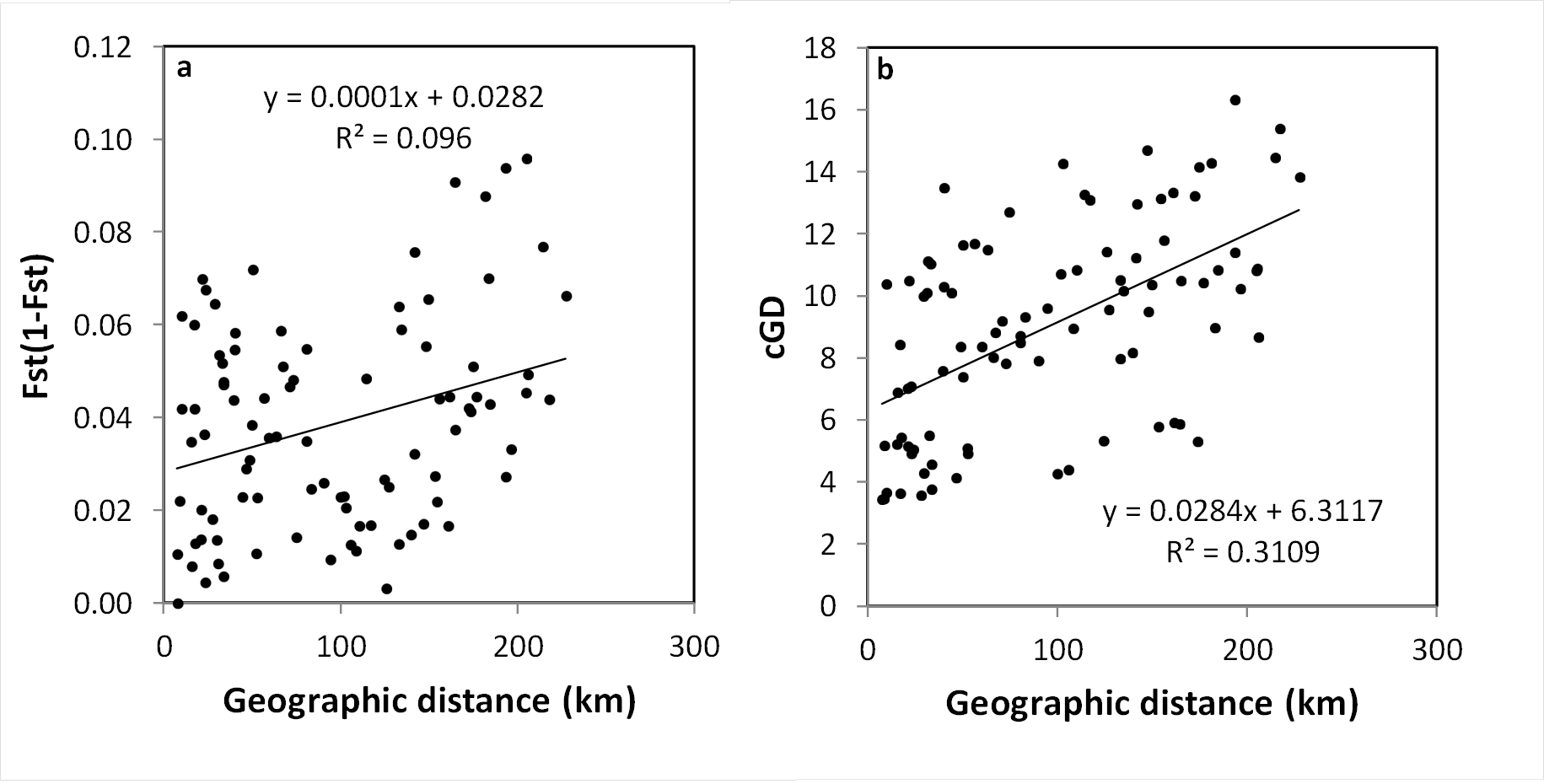


A

B

Supplement: S2 Fig — (DOCX) [file pone.0194901.s002.docx]

**S3 Fig**


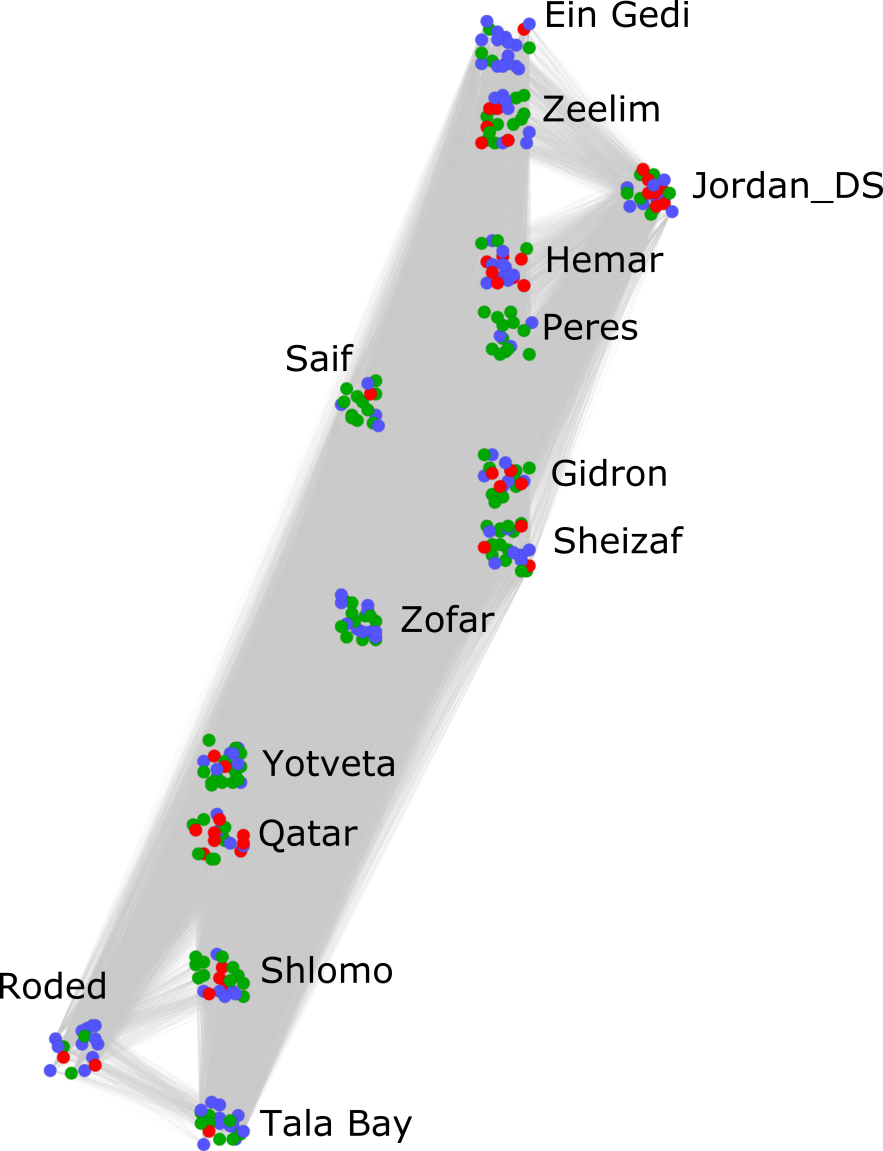

Supplement: S3 Fig — Nodes represent individual trees, with positions approximately corresponding to sampling sites; edges, in gray, represent genetic similarity. Node colors indicate the detected communities using NetStruct, which are determined independently of sampling site and without any a priori assumptions or underlying model. The distribution of communities in the population is not independent of sampling sites, with a rejection of the null hypothesis at p-value < 0.0001 (see S4 Table). (DOCX) [file pone.0194901.s003.docx]
