# Supplementary material for: Detecting hierarchical levels of connectivity in a population of Acacia tortilis at the northern edge of the species’ global distribution: Combining classical population genetics and network analyses
Source: PLoS One. 2018 Apr 12;13(4):e0194901. doi: 10.1371/journal.pone.0194901 (PMC5896914; doi:10.1371/journal.pone.0194901)
Supplement: S1 Table — (DOCX) [file pone.0194901.s004.docx]

**S1 Table**

| **Locus** | **Allele/n** | **Ein Gedi** | **Zeelim** | **Jordan_DS** | **Hemar** | **Peres** | **Saif** | **Gidron** | **Sheizaf** | **Zofar** | **Yotveta** | **Qatar_J** | **Roded** | **Shlomo** | **Tala Bay** | **Egypt** | **Sudan** |
| --- | --- | --- | --- | --- | --- | --- | --- | --- | --- | --- | --- | --- | --- | --- | --- | --- | --- |
| **L2** | **N** | 21 | 24 | 24 | 19 | 11 | 15 | 19 | 22 | 21 | 18 | 20 | 20 | 18 | 23 | 9 | 12 |
|  | **152** | 0.00 | 0.00 | 0.00 | 0.00 | 0.00 | 0.00 | 0.00 | 0.00 | 0.00 | 0.00 | 0.03 | 0.00 | 0.00 | 0.00 | 0.39 | 0.08 |
|  | **154** | 0.00 | 0.00 | 0.00 | 0.00 | 0.00 | 0.00 | 0.00 | 0.00 | 0.00 | 0.00 | 0.00 | 0.00 | 0.00 | 0.00 | 0.33 | 0.04 |
|  | **156** | 0.00 | 0.00 | 0.00 | 0.00 | 0.00 | 0.00 | 0.00 | 0.00 | 0.00 | 0.00 | 0.00 | 0.00 | 0.00 | 0.00 | 0.06 | 0.00 |
|  | **158** | 0.00 | 0.06 | 0.02 | 0.11 | 0.14 | 0.13 | 0.00 | 0.09 | 0.00 | 0.11 | 0.03 | 0.00 | 0.00 | 0.07 | 0.11 | 0.00 |
|  | **160** | 0.00 | 0.00 | 0.15 | 0.00 | 0.00 | 0.00 | 0.00 | 0.00 | 0.00 | 0.00 | 0.13 | 0.00 | 0.00 | 0.00 | 0.00 | 0.00 |
|  | **162** | 0.00 | 0.00 | 0.04 | 0.00 | 0.00 | 0.00 | 0.00 | 0.00 | 0.00 | 0.03 | 0.00 | 0.00 | 0.00 | 0.02 | 0.06 | 0.29 |
|  | **164** | 0.24 | 0.38 | 0.42 | 0.39 | 0.55 | 0.30 | 0.34 | 0.43 | 0.24 | 0.47 | 0.38 | 0.30 | 0.44 | 0.48 | 0.00 | 0.00 |
|  | **166** | 0.02 | 0.00 | 0.02 | 0.05 | 0.00 | 0.07 | 0.05 | 0.18 | 0.19 | 0.08 | 0.10 | 0.20 | 0.06 | 0.09 | 0.00 | 0.13 |
|  | **168** | 0.00 | 0.00 | 0.00 | 0.00 | 0.00 | 0.00 | 0.00 | 0.00 | 0.00 | 0.00 | 0.05 | 0.00 | 0.06 | 0.02 | 0.06 | 0.04 |
|  | **170** | 0.00 | 0.00 | 0.00 | 0.00 | 0.00 | 0.00 | 0.00 | 0.00 | 0.00 | 0.00 | 0.00 | 0.00 | 0.00 | 0.00 | 0.00 | 0.04 |
|  | **172** | 0.00 | 0.00 | 0.00 | 0.00 | 0.00 | 0.00 | 0.00 | 0.00 | 0.00 | 0.00 | 0.03 | 0.00 | 0.00 | 0.00 | 0.00 | 0.04 |
|  | **174** | 0.00 | 0.00 | 0.02 | 0.03 | 0.00 | 0.00 | 0.00 | 0.00 | 0.00 | 0.00 | 0.00 | 0.00 | 0.00 | 0.00 | 0.00 | 0.00 |
|  | **176** | 0.00 | 0.00 | 0.00 | 0.00 | 0.00 | 0.00 | 0.00 | 0.00 | 0.00 | 0.08 | 0.00 | 0.00 | 0.00 | 0.00 | 0.00 | 0.00 |
|  | **178** | 0.24 | 0.02 | 0.10 | 0.08 | 0.09 | 0.17 | 0.16 | 0.07 | 0.19 | 0.14 | 0.08 | 0.03 | 0.08 | 0.11 | 0.00 | 0.08 |
|  | **180** | 0.05 | 0.10 | 0.02 | 0.00 | 0.05 | 0.10 | 0.13 | 0.05 | 0.07 | 0.03 | 0.10 | 0.23 | 0.14 | 0.00 | 0.00 | 0.08 |
|  | **182** | 0.00 | 0.02 | 0.04 | 0.11 | 0.05 | 0.13 | 0.03 | 0.07 | 0.02 | 0.03 | 0.03 | 0.10 | 0.14 | 0.17 | 0.00 | 0.04 |
|  | **184** | 0.10 | 0.04 | 0.08 | 0.08 | 0.09 | 0.00 | 0.03 | 0.07 | 0.02 | 0.00 | 0.03 | 0.05 | 0.03 | 0.02 | 0.00 | 0.04 |
|  | **186** | 0.31 | 0.35 | 0.04 | 0.11 | 0.05 | 0.10 | 0.13 | 0.02 | 0.21 | 0.03 | 0.03 | 0.00 | 0.03 | 0.00 | 0.00 | 0.00 |
|  | **188** | 0.02 | 0.02 | 0.02 | 0.05 | 0.00 | 0.00 | 0.05 | 0.02 | 0.05 | 0.00 | 0.03 | 0.05 | 0.00 | 0.00 | 0.00 | 0.08 |
|  | **190** | 0.00 | 0.00 | 0.02 | 0.00 | 0.00 | 0.00 | 0.05 | 0.00 | 0.00 | 0.00 | 0.00 | 0.00 | 0.00 | 0.00 | 0.00 | 0.00 |
|  | **192** | 0.02 | 0.00 | 0.00 | 0.00 | 0.00 | 0.00 | 0.00 | 0.00 | 0.00 | 0.00 | 0.00 | 0.00 | 0.00 | 0.00 | 0.00 | 0.00 |
|  | **194** | 0.00 | 0.00 | 0.00 | 0.00 | 0.00 | 0.00 | 0.03 | 0.00 | 0.00 | 0.00 | 0.00 | 0.00 | 0.03 | 0.02 | 0.00 | 0.00 |
|  | **198** | 0.00 | 0.00 | 0.00 | 0.00 | 0.00 | 0.00 | 0.00 | 0.00 | 0.00 | 0.00 | 0.00 | 0.03 | 0.00 | 0.00 | 0.00 | 0.00 |
|  | **200** | 0.00 | 0.00 | 0.00 | 0.00 | 0.00 | 0.00 | 0.00 | 0.00 | 0.00 | 0.00 | 0.00 | 0.03 | 0.00 | 0.00 | 0.00 | 0.00 |
| **L3** | **N** | 21 | 24 | 20 | 20 | 15 | 16 | 20 | 22 | 21 | 23 | 20 | 21 | 18 | 22 | 14 | 12 |
|  | **63** | 0.00 | 0.00 | 0.00 | 0.00 | 0.00 | 0.00 | 0.00 | 0.00 | 0.00 | 0.00 | 0.00 | 0.00 | 0.00 | 0.00 | 0.04 | 0.00 |
|  | **75** | 0.00 | 0.00 | 0.00 | 0.00 | 0.00 | 0.00 | 0.00 | 0.00 | 0.00 | 0.00 | 0.00 | 0.00 | 0.00 | 0.00 | 0.04 | 0.00 |
|  | **83** | 0.00 | 0.00 | 0.00 | 0.00 | 0.00 | 0.00 | 0.00 | 0.00 | 0.00 | 0.02 | 0.00 | 0.02 | 0.00 | 0.00 | 0.43 | 0.00 |
|  | **85** | 0.17 | 0.42 | 0.25 | 0.15 | 0.13 | 0.00 | 0.15 | 0.02 | 0.10 | 0.15 | 0.20 | 0.21 | 0.06 | 0.18 | 0.32 | 0.13 |
|  | **87** | 0.33 | 0.31 | 0.30 | 0.40 | 0.20 | 0.38 | 0.23 | 0.34 | 0.50 | 0.52 | 0.45 | 0.40 | 0.69 | 0.50 | 0.07 | 0.42 |
|  | **89** | 0.10 | 0.02 | 0.15 | 0.15 | 0.17 | 0.16 | 0.30 | 0.18 | 0.02 | 0.00 | 0.08 | 0.05 | 0.11 | 0.11 | 0.07 | 0.04 |
|  | **91** | 0.00 | 0.00 | 0.03 | 0.10 | 0.00 | 0.03 | 0.00 | 0.00 | 0.05 | 0.00 | 0.03 | 0.00 | 0.00 | 0.00 | 0.04 | 0.17 |
|  | **93** | 0.31 | 0.15 | 0.25 | 0.15 | 0.33 | 0.22 | 0.18 | 0.30 | 0.29 | 0.22 | 0.08 | 0.14 | 0.03 | 0.07 | 0.00 | 0.04 |
|  | **95** | 0.10 | 0.10 | 0.00 | 0.05 | 0.13 | 0.16 | 0.13 | 0.14 | 0.05 | 0.09 | 0.13 | 0.17 | 0.06 | 0.11 | 0.00 | 0.00 |
|  | **97** | 0.00 | 0.00 | 0.00 | 0.00 | 0.00 | 0.03 | 0.00 | 0.02 | 0.00 | 0.00 | 0.03 | 0.00 | 0.06 | 0.02 | 0.00 | 0.00 |
|  | **99** | 0.00 | 0.00 | 0.03 | 0.00 | 0.03 | 0.03 | 0.03 | 0.00 | 0.00 | 0.00 | 0.00 | 0.00 | 0.00 | 0.00 | 0.00 | 0.00 |
|  | **101** | 0.00 | 0.00 | 0.00 | 0.00 | 0.00 | 0.00 | 0.00 | 0.00 | 0.00 | 0.00 | 0.00 | 0.00 | 0.00 | 0.00 | 0.00 | 0.13 |
|  | **103** | 0.00 | 0.00 | 0.00 | 0.00 | 0.00 | 0.00 | 0.00 | 0.00 | 0.00 | 0.00 | 0.00 | 0.00 | 0.00 | 0.00 | 0.00 | 0.04 |
|  | **105** | 0.00 | 0.00 | 0.00 | 0.00 | 0.00 | 0.00 | 0.00 | 0.00 | 0.00 | 0.00 | 0.00 | 0.00 | 0.00 | 0.00 | 0.00 | 0.04 |
|  | **107** | 0.00 | 0.00 | 0.00 | 0.00 | 0.00 | 0.00 | 0.00 | 0.00 | 0.00 | 0.00 | 0.03 | 0.00 | 0.00 | 0.00 | 0.00 | 0.00 |
| **L6** | **N** | 21 | 22 | 23 | 19 | 14 | 17 | 18 | 24 | 19 | 22 | 20 | 20 | 18 | 21 | 14 | 12 |
|  | **78** | 0.57 | 0.50 | 0.43 | 0.16 | 0.32 | 0.47 | 0.56 | 0.33 | 0.53 | 0.32 | 0.48 | 0.33 | 0.67 | 0.36 | 0.50 | 0.08 |
|  | **80** | 0.00 | 0.00 | 0.00 | 0.00 | 0.00 | 0.00 | 0.00 | 0.00 | 0.00 | 0.00 | 0.00 | 0.00 | 0.00 | 0.02 | 0.00 | 0.00 |
|  | **82** | 0.00 | 0.00 | 0.00 | 0.00 | 0.00 | 0.00 | 0.00 | 0.00 | 0.00 | 0.00 | 0.00 | 0.00 | 0.00 | 0.00 | 0.07 | 0.00 |
|  | **84** | 0.00 | 0.00 | 0.00 | 0.00 | 0.00 | 0.00 | 0.00 | 0.00 | 0.00 | 0.00 | 0.00 | 0.00 | 0.00 | 0.00 | 0.18 | 0.00 |
|  | **90** | 0.00 | 0.00 | 0.00 | 0.00 | 0.00 | 0.00 | 0.00 | 0.00 | 0.00 | 0.00 | 0.00 | 0.00 | 0.00 | 0.00 | 0.00 | 0.38 |
|  | **92** | 0.00 | 0.00 | 0.00 | 0.00 | 0.00 | 0.00 | 0.00 | 0.00 | 0.00 | 0.07 | 0.00 | 0.00 | 0.00 | 0.00 | 0.00 | 0.04 |
|  | **94** | 0.00 | 0.05 | 0.04 | 0.05 | 0.00 | 0.03 | 0.03 | 0.02 | 0.03 | 0.09 | 0.03 | 0.08 | 0.11 | 0.02 | 0.00 | 0.00 |
|  | **96** | 0.00 | 0.00 | 0.13 | 0.05 | 0.00 | 0.06 | 0.03 | 0.04 | 0.08 | 0.09 | 0.08 | 0.03 | 0.00 | 0.05 | 0.00 | 0.00 |
|  | **98** | 0.00 | 0.00 | 0.00 | 0.00 | 0.04 | 0.06 | 0.00 | 0.04 | 0.00 | 0.00 | 0.00 | 0.00 | 0.00 | 0.10 | 0.04 | 0.04 |
|  | **100** | 0.02 | 0.02 | 0.24 | 0.18 | 0.29 | 0.18 | 0.11 | 0.13 | 0.03 | 0.11 | 0.08 | 0.08 | 0.03 | 0.07 | 0.00 | 0.25 |
|  | **102** | 0.05 | 0.09 | 0.07 | 0.11 | 0.07 | 0.03 | 0.00 | 0.10 | 0.00 | 0.07 | 0.05 | 0.03 | 0.11 | 0.10 | 0.07 | 0.00 |
|  | **104** | 0.26 | 0.23 | 0.02 | 0.08 | 0.11 | 0.06 | 0.03 | 0.04 | 0.16 | 0.05 | 0.08 | 0.10 | 0.00 | 0.05 | 0.07 | 0.04 |
|  | **106** | 0.07 | 0.05 | 0.02 | 0.21 | 0.11 | 0.03 | 0.14 | 0.15 | 0.05 | 0.14 | 0.15 | 0.13 | 0.08 | 0.14 | 0.00 | 0.00 |
|  | **108** | 0.02 | 0.02 | 0.02 | 0.08 | 0.00 | 0.06 | 0.11 | 0.10 | 0.13 | 0.07 | 0.08 | 0.20 | 0.00 | 0.10 | 0.00 | 0.13 |
|  | **110** | 0.00 | 0.00 | 0.02 | 0.08 | 0.07 | 0.03 | 0.00 | 0.04 | 0.00 | 0.00 | 0.00 | 0.05 | 0.00 | 0.00 | 0.00 | 0.04 |
|  | **112** | 0.00 | 0.05 | 0.00 | 0.00 | 0.00 | 0.00 | 0.00 | 0.00 | 0.00 | 0.00 | 0.00 | 0.00 | 0.00 | 0.00 | 0.00 | 0.00 |
|  | **114** | 0.00 | 0.00 | 0.00 | 0.00 | 0.00 | 0.00 | 0.00 | 0.00 | 0.00 | 0.00 | 0.00 | 0.00 | 0.00 | 0.00 | 0.07 | 0.00 |
| **L8** | **N** | 21 | 24 | 23 | 20 | 15 | 17 | 20 | 24 | 21 | 23 | 17 | 21 | 18 | 24 | 13 | 12 |
|  | **166** | 0.00 | 0.00 | 0.00 | 0.00 | 0.00 | 0.00 | 0.00 | 0.00 | 0.00 | 0.02 | 0.00 | 0.00 | 0.00 | 0.00 | 0.00 | 0.00 |
|  | **168** | 0.00 | 0.00 | 0.00 | 0.00 | 0.00 | 0.00 | 0.00 | 0.00 | 0.00 | 0.00 | 0.00 | 0.00 | 0.00 | 0.00 | 0.38 | 0.00 |
|  | **170** | 0.14 | 0.25 | 0.13 | 0.08 | 0.50 | 0.29 | 0.18 | 0.23 | 0.29 | 0.24 | 0.03 | 0.17 | 0.14 | 0.13 | 0.15 | 0.08 |
|  | **172** | 0.00 | 0.00 | 0.00 | 0.00 | 0.00 | 0.00 | 0.00 | 0.00 | 0.02 | 0.00 | 0.00 | 0.00 | 0.00 | 0.00 | 0.00 | 0.00 |
|  | **174** | 0.00 | 0.00 | 0.09 | 0.00 | 0.03 | 0.03 | 0.00 | 0.04 | 0.00 | 0.02 | 0.00 | 0.02 | 0.06 | 0.00 | 0.00 | 0.08 |
|  | **176** | 0.00 | 0.00 | 0.00 | 0.00 | 0.00 | 0.00 | 0.00 | 0.00 | 0.00 | 0.00 | 0.00 | 0.00 | 0.00 | 0.00 | 0.35 | 0.08 |
|  | **178** | 0.00 | 0.00 | 0.00 | 0.03 | 0.00 | 0.00 | 0.00 | 0.00 | 0.00 | 0.00 | 0.00 | 0.00 | 0.00 | 0.00 | 0.12 | 0.00 |
|  | **180** | 0.00 | 0.00 | 0.00 | 0.00 | 0.00 | 0.00 | 0.03 | 0.02 | 0.00 | 0.02 | 0.00 | 0.00 | 0.00 | 0.00 | 0.00 | 0.29 |
|  | **182** | 0.02 | 0.19 | 0.07 | 0.23 | 0.03 | 0.09 | 0.08 | 0.02 | 0.05 | 0.04 | 0.09 | 0.07 | 0.00 | 0.02 | 0.00 | 0.17 |
|  | **184** | 0.55 | 0.29 | 0.22 | 0.48 | 0.30 | 0.15 | 0.20 | 0.31 | 0.29 | 0.28 | 0.09 | 0.19 | 0.58 | 0.38 | 0.00 | 0.08 |
|  | **186** | 0.12 | 0.15 | 0.28 | 0.15 | 0.10 | 0.35 | 0.40 | 0.23 | 0.21 | 0.24 | 0.38 | 0.29 | 0.11 | 0.19 | 0.00 | 0.00 |
|  | **188** | 0.00 | 0.02 | 0.07 | 0.00 | 0.00 | 0.00 | 0.00 | 0.04 | 0.00 | 0.07 | 0.09 | 0.00 | 0.00 | 0.04 | 0.00 | 0.17 |
|  | **190** | 0.00 | 0.00 | 0.00 | 0.00 | 0.00 | 0.00 | 0.00 | 0.02 | 0.02 | 0.00 | 0.00 | 0.00 | 0.00 | 0.00 | 0.00 | 0.04 |
|  | **192** | 0.00 | 0.00 | 0.00 | 0.00 | 0.03 | 0.00 | 0.03 | 0.00 | 0.07 | 0.00 | 0.06 | 0.02 | 0.00 | 0.00 | 0.00 | 0.00 |
|  | **194** | 0.00 | 0.00 | 0.00 | 0.00 | 0.00 | 0.00 | 0.00 | 0.00 | 0.00 | 0.02 | 0.00 | 0.00 | 0.00 | 0.00 | 0.00 | 0.00 |
|  | **200** | 0.00 | 0.00 | 0.00 | 0.00 | 0.00 | 0.00 | 0.00 | 0.00 | 0.00 | 0.00 | 0.00 | 0.02 | 0.00 | 0.02 | 0.00 | 0.00 |
|  | **202** | 0.02 | 0.06 | 0.04 | 0.03 | 0.00 | 0.00 | 0.00 | 0.00 | 0.00 | 0.00 | 0.00 | 0.00 | 0.00 | 0.00 | 0.00 | 0.00 |
|  | **204** | 0.00 | 0.00 | 0.00 | 0.03 | 0.00 | 0.00 | 0.00 | 0.00 | 0.00 | 0.00 | 0.00 | 0.00 | 0.00 | 0.02 | 0.00 | 0.00 |
|  | **206** | 0.00 | 0.00 | 0.00 | 0.00 | 0.00 | 0.00 | 0.00 | 0.00 | 0.00 | 0.00 | 0.00 | 0.05 | 0.00 | 0.00 | 0.00 | 0.00 |
|  | **208** | 0.00 | 0.00 | 0.02 | 0.00 | 0.00 | 0.00 | 0.03 | 0.00 | 0.00 | 0.00 | 0.00 | 0.05 | 0.00 | 0.06 | 0.00 | 0.00 |
|  | **210** | 0.07 | 0.04 | 0.02 | 0.00 | 0.00 | 0.03 | 0.00 | 0.00 | 0.00 | 0.00 | 0.03 | 0.05 | 0.00 | 0.02 | 0.00 | 0.00 |
|  | **212** | 0.05 | 0.00 | 0.04 | 0.00 | 0.00 | 0.03 | 0.03 | 0.02 | 0.05 | 0.00 | 0.03 | 0.02 | 0.00 | 0.04 | 0.00 | 0.00 |
|  | **214** | 0.02 | 0.00 | 0.02 | 0.00 | 0.00 | 0.00 | 0.00 | 0.04 | 0.00 | 0.02 | 0.21 | 0.02 | 0.08 | 0.00 | 0.00 | 0.00 |
|  | **216** | 0.00 | 0.00 | 0.00 | 0.00 | 0.00 | 0.03 | 0.03 | 0.00 | 0.00 | 0.00 | 0.00 | 0.02 | 0.00 | 0.02 | 0.00 | 0.00 |
|  | **218** | 0.00 | 0.00 | 0.00 | 0.00 | 0.00 | 0.00 | 0.03 | 0.02 | 0.00 | 0.00 | 0.00 | 0.00 | 0.03 | 0.06 | 0.00 | 0.00 |
|  | **220** | 0.00 | 0.00 | 0.00 | 0.00 | 0.00 | 0.00 | 0.00 | 0.00 | 0.00 | 0.02 | 0.00 | 0.00 | 0.00 | 0.00 | 0.00 | 0.00 |
| **L10** | **N** | 21 | 24 | 23 | 18 | 15 | 17 | 20 | 24 | 21 | 22 | 20 | 21 | 18 | 24 | 14 | 12 |
|  | **78** | 0.00 | 0.00 | 0.00 | 0.00 | 0.00 | 0.00 | 0.00 | 0.00 | 0.00 | 0.00 | 0.00 | 0.00 | 0.00 | 0.00 | 0.00 | 0.04 |
|  | **80** | 0.00 | 0.00 | 0.00 | 0.00 | 0.00 | 0.00 | 0.00 | 0.00 | 0.00 | 0.00 | 0.03 | 0.00 | 0.00 | 0.02 | 0.54 | 0.04 |
|  | **82** | 0.00 | 0.00 | 0.00 | 0.00 | 0.00 | 0.00 | 0.00 | 0.00 | 0.00 | 0.00 | 0.00 | 0.00 | 0.00 | 0.06 | 0.29 | 0.04 |
|  | **84** | 0.00 | 0.00 | 0.00 | 0.00 | 0.00 | 0.00 | 0.00 | 0.00 | 0.00 | 0.00 | 0.00 | 0.00 | 0.00 | 0.02 | 0.00 | 0.00 |
|  | **86** | 0.00 | 0.00 | 0.00 | 0.00 | 0.00 | 0.00 | 0.00 | 0.00 | 0.00 | 0.00 | 0.00 | 0.00 | 0.03 | 0.00 | 0.00 | 0.04 |
|  | **88** | 0.00 | 0.00 | 0.00 | 0.00 | 0.00 | 0.00 | 0.00 | 0.00 | 0.00 | 0.00 | 0.10 | 0.00 | 0.00 | 0.02 | 0.18 | 0.00 |
|  | **90** | 0.17 | 0.38 | 0.17 | 0.44 | 0.37 | 0.47 | 0.38 | 0.25 | 0.38 | 0.27 | 0.33 | 0.24 | 0.39 | 0.48 | 0.00 | 0.00 |
|  | **92** | 0.05 | 0.04 | 0.07 | 0.00 | 0.00 | 0.06 | 0.10 | 0.08 | 0.00 | 0.11 | 0.05 | 0.07 | 0.00 | 0.10 | 0.00 | 0.33 |
|  | **94** | 0.26 | 0.08 | 0.28 | 0.19 | 0.37 | 0.26 | 0.15 | 0.23 | 0.24 | 0.16 | 0.23 | 0.24 | 0.39 | 0.06 | 0.00 | 0.08 |
|  | **96** | 0.19 | 0.19 | 0.13 | 0.08 | 0.07 | 0.06 | 0.23 | 0.25 | 0.14 | 0.16 | 0.20 | 0.29 | 0.08 | 0.10 | 0.00 | 0.29 |
|  | **98** | 0.29 | 0.27 | 0.24 | 0.22 | 0.20 | 0.12 | 0.13 | 0.17 | 0.19 | 0.27 | 0.08 | 0.14 | 0.03 | 0.10 | 0.00 | 0.04 |
|  | **100** | 0.05 | 0.04 | 0.02 | 0.06 | 0.00 | 0.00 | 0.00 | 0.00 | 0.00 | 0.00 | 0.00 | 0.00 | 0.00 | 0.00 | 0.00 | 0.08 |
|  | **102** | 0.00 | 0.00 | 0.00 | 0.00 | 0.00 | 0.00 | 0.00 | 0.00 | 0.02 | 0.00 | 0.00 | 0.00 | 0.00 | 0.00 | 0.00 | 0.00 |
|  | **104** | 0.00 | 0.00 | 0.00 | 0.00 | 0.00 | 0.00 | 0.00 | 0.00 | 0.00 | 0.00 | 0.00 | 0.02 | 0.06 | 0.00 | 0.00 | 0.00 |
|  | **106** | 0.00 | 0.00 | 0.09 | 0.00 | 0.00 | 0.03 | 0.03 | 0.02 | 0.02 | 0.02 | 0.00 | 0.00 | 0.00 | 0.02 | 0.00 | 0.00 |
|  | **118** | 0.00 | 0.00 | 0.00 | 0.00 | 0.00 | 0.00 | 0.00 | 0.00 | 0.00 | 0.00 | 0.00 | 0.00 | 0.03 | 0.00 | 0.00 | 0.00 |
| **L11** | **N** | 20 | 24 | 22 | 20 | 15 | 17 | 20 | 24 | 21 | 23 | 20 | 21 | 17 | 24 | 14 | 12 |
|  | **100** | 0.00 | 0.00 | 0.09 | 0.00 | 0.00 | 0.00 | 0.00 | 0.00 | 0.00 | 0.00 | 0.00 | 0.00 | 0.00 | 0.00 | 0.04 | 0.00 |
|  | **106** | 0.00 | 0.06 | 0.39 | 0.15 | 0.13 | 0.12 | 0.20 | 0.06 | 0.02 | 0.13 | 0.03 | 0.02 | 0.00 | 0.04 | 0.04 | 0.00 |
|  | **108** | 0.00 | 0.00 | 0.00 | 0.00 | 0.00 | 0.00 | 0.00 | 0.00 | 0.00 | 0.02 | 0.08 | 0.02 | 0.12 | 0.02 | 0.07 | 0.00 |
|  | **110** | 0.00 | 0.00 | 0.02 | 0.00 | 0.00 | 0.03 | 0.00 | 0.00 | 0.02 | 0.04 | 0.00 | 0.05 | 0.06 | 0.06 | 0.07 | 0.17 |
|  | **112** | 0.38 | 0.04 | 0.09 | 0.43 | 0.07 | 0.06 | 0.13 | 0.23 | 0.12 | 0.02 | 0.05 | 0.12 | 0.00 | 0.06 | 0.18 | 0.08 |
|  | **114** | 0.03 | 0.08 | 0.11 | 0.05 | 0.07 | 0.09 | 0.13 | 0.04 | 0.14 | 0.09 | 0.05 | 0.00 | 0.06 | 0.13 | 0.11 | 0.08 |
|  | **116** | 0.13 | 0.02 | 0.07 | 0.08 | 0.00 | 0.03 | 0.00 | 0.04 | 0.07 | 0.13 | 0.05 | 0.10 | 0.12 | 0.08 | 0.04 | 0.08 |
|  | **118** | 0.35 | 0.54 | 0.09 | 0.15 | 0.47 | 0.38 | 0.35 | 0.48 | 0.48 | 0.33 | 0.48 | 0.43 | 0.38 | 0.33 | 0.29 | 0.00 |
|  | **120** | 0.03 | 0.04 | 0.09 | 0.08 | 0.00 | 0.12 | 0.10 | 0.00 | 0.07 | 0.07 | 0.03 | 0.07 | 0.03 | 0.08 | 0.11 | 0.21 |
|  | **122** | 0.03 | 0.04 | 0.02 | 0.05 | 0.00 | 0.00 | 0.00 | 0.02 | 0.00 | 0.00 | 0.00 | 0.05 | 0.00 | 0.00 | 0.04 | 0.00 |
|  | **124** | 0.03 | 0.00 | 0.02 | 0.00 | 0.27 | 0.12 | 0.08 | 0.10 | 0.07 | 0.04 | 0.13 | 0.05 | 0.24 | 0.17 | 0.00 | 0.13 |
|  | **126** | 0.05 | 0.17 | 0.00 | 0.00 | 0.00 | 0.06 | 0.03 | 0.02 | 0.00 | 0.07 | 0.10 | 0.07 | 0.00 | 0.02 | 0.04 | 0.13 |
|  | **128** | 0.00 | 0.00 | 0.00 | 0.00 | 0.00 | 0.00 | 0.00 | 0.00 | 0.00 | 0.07 | 0.03 | 0.02 | 0.00 | 0.00 | 0.00 | 0.08 |
|  | **130** | 0.00 | 0.00 | 0.00 | 0.00 | 0.00 | 0.00 | 0.00 | 0.00 | 0.00 | 0.00 | 0.00 | 0.00 | 0.00 | 0.00 | 0.00 | 0.04 |
|  | **132** | 0.00 | 0.00 | 0.00 | 0.03 | 0.00 | 0.00 | 0.00 | 0.00 | 0.00 | 0.00 | 0.00 | 0.00 | 0.00 | 0.00 | 0.00 | 0.00 |
