# Supplementary material for: Detecting hierarchical levels of connectivity in a population of Acacia tortilis at the northern edge of the species’ global distribution: Combining classical population genetics and network analyses
Source: PLoS One. 2018 Apr 12;13(4):e0194901. doi: 10.1371/journal.pone.0194901 (PMC5896914; doi:10.1371/journal.pone.0194901)
Supplement: S2 Table — (DOCX) [file pone.0194901.s005.docx]

**S2 Table**

| **Primer name** | **Locus** | **Primer sequence (5' - 3')** | **Allele size range (bp)** | **Fluorescent dye** |
| --- | --- | --- | --- | --- |
| L2 | Acator_01934 | F CACATTTAACTCGCTTTGGCG | 150-195 | PET |
|  |  | R CACATATGGCTGCTGTGGAG |  |  |
| L3 | Acator_15865 | F CCTCTTGGTTGTTGATGTAAAACAG | 100-150 | FAM |
|  |  | R TCGAAGAAGCTACAATGGCTG |  |  |
| L5 | Acator_25711 | F TCAATGACCCCCTAAATAAGAGC | 85-100 | NED |
|  |  | R GGTTAGGACCTCAAACGTGC |  |  |
| L6 | Acator_22993 | F CATCGGCCCAGTTATGAGTTG | 75-110 | PET |
|  |  | R TCCAAAGTTCTGATACTGGTAAGC |  |  |
| L8 | Acator_23023 | F TGGCTTTGTTTCGATGGCAG | 170-215 | FAM |
|  |  | R TCGTTGAAGATGTGCTATGTGTC |  |  |
| L9 | Acator_15039 | F CGCAAAGTTGCATGTGATCTTC | 100-315 | NED |
|  |  | R ACTAGCTACCATCAAACGTGC |  |  |
| L10 | Acator_03250 | F TGCGAGTGTCTGATAACCAC | 90-105 | VIC |
|  |  | R AGGCAATATGCAAGCTCCAC |  |  |
| L11 | Acator_15563 | F TCGTACTCATCGCCAAGACC | 105-130 | VIC |
|  |  | R CGAAGAAGCTACAATGGCTG |  |  |
