# Supplementary material for: Detecting hierarchical levels of connectivity in a population of Acacia tortilis at the northern edge of the species’ global distribution: Combining classical population genetics and network analyses
Source: PLoS One. 2018 Apr 12;13(4):e0194901. doi: 10.1371/journal.pone.0194901 (PMC5896914; doi:10.1371/journal.pone.0194901)
Supplement: S3 Table — Fst values are below the diagonal; p-values based on 999 permutations are shown above the diagonal. (DOCX) [file pone.0194901.s006.docx]

**S3 Table**

|  | **EG** | **ZE** | **J** | **H** | **P** | **SF** | **G** | **SH** | **ZF** | **Y** | **Q** | **R** | **SO** | **TB** |
| --- | --- | --- | --- | --- | --- | --- | --- | --- | --- | --- | --- | --- | --- | --- |
| **EG** | *** | 0.001 | 0.001 | 0.001 | 0.001 | 0.001 | 0.001 | 0.001 | 0.015 | 0.001 | 0.001 | 0.001 | 0.001 | 0.001 |
| **ZE** | 0.041 | *** | 0.001 | 0.001 | 0.001 | 0.002 | 0.001 | 0.001 | 0.008 | 0.001 | 0.001 | 0.004 | 0.001 | 0.001 |
| **J** | 0.059 | 0.065 | *** | 0.001 | 0.001 | 0.002 | 0.010 | 0.001 | 0.001 | 0.001 | 0.001 | 0.001 | 0.001 | 0.001 |
| **H** | 0.045 | 0.064 | 0.038 | *** | 0.001 | 0.001 | 0.001 | 0.001 | 0.001 | 0.001 | 0.001 | 0.001 | 0.001 | 0.004 |
| **P** | 0.065 | 0.055 | 0.048 | 0.054 | *** | 0.119 | 0.004 | 0.099 | 0.004 | 0.010 | 0.001 | 0.001 | 0.001 | 0.001 |
| **SF** | 0.055 | 0.044 | 0.029 | 0.044 | 0.011 | *** | 0.466 | 0.161 | 0.184 | 0.086 | 0.034 | 0.046 | 0.001 | 0.031 |
| **G** | 0.045 | 0.035 | 0.020 | 0.040 | 0.032 | 0.000 | *** | 0.094 | 0.026 | 0.006 | 0.022 | 0.069 | 0.001 | 0.006 |
| **SH** | 0.033 | 0.045 | 0.033 | 0.027 | 0.011 | 0.007 | 0.009 | *** | 0.032 | 0.079 | 0.007 | 0.350 | 0.001 | 0.019 |
| **ZF** | 0.023 | 0.026 | 0.051 | 0.048 | 0.036 | 0.006 | 0.017 | 0.013 | *** | 0.035 | 0.005 | 0.065 | 0.001 | 0.003 |
| **Y** | 0.048 | 0.036 | 0.025 | 0.030 | 0.024 | 0.011 | 0.021 | 0.008 | 0.014 | *** | 0.005 | 0.148 | 0.001 | 0.070 |
| **Q** | 0.064 | 0.039 | 0.040 | 0.060 | 0.057 | 0.015 | 0.014 | 0.019 | 0.023 | 0.020 | *** | 0.347 | 0.001 | 0.007 |
| **R** | 0.045 | 0.032 | 0.039 | 0.038 | 0.039 | 0.013 | 0.010 | 0.001 | 0.011 | 0.007 | 0.002 | *** | 0.001 | 0.008 |
| **SO** | 0.070 | 0.088 | 0.084 | 0.079 | 0.080 | 0.052 | 0.068 | 0.054 | 0.046 | 0.051 | 0.047 | 0.056 | *** | 0.002 |
| **TB** | 0.061 | 0.042 | 0.042 | 0.025 | 0.040 | 0.015 | 0.019 | 0.015 | 0.024 | 0.009 | 0.020 | 0.018 | 0.032 | *** |
| Subpopulation Code: EG, Ein Gedi; ZE, Zeelim; J, Jordan_DS; H, Hemar; P, Peres; SF, Saif; G, Gidron; SH, Sheizaf; ZF, Zofar; Y, Yotveta; Q, Qatar; R, Roded; SO, Shlomo; TB, Tala Bay | | | | | | | | | | | | | | |
