# Supplementary material for: Detecting hierarchical levels of connectivity in a population of Acacia tortilis at the northern edge of the species’ global distribution: Combining classical population genetics and network analyses
Source: PLoS One. 2018 Apr 12;13(4):e0194901. doi: 10.1371/journal.pone.0194901 (PMC5896914; doi:10.1371/journal.pone.0194901)
Supplement: S1 Text — (DOCX) [file pone.0194901.s008.docx]

**S1 Text**

**NetStruct Analysis**

A full account of the *NetStruct* method appears in Greenabum et al. (2016), and the *NetStruct* software can be downloaded at <https://giligreenbaum.wordpress.com/software>. The method is composed of three steps, with a fourth hypothesis-testing step implemented in this paper:

*Step I: Constructing a genetic similarity network*

A pairwise genetic similarity matrix between all pairs of individuals is constructed. The genetic similarity measure between each pair that is implemented in *NetStruct* is a frequency-weighted allele-sharing measure. This measure incorporates the genotypes of the individuals, as well as the frequency (in the entire sample) of the alleles they share, in which individuals that share few alleles or common alleles will have lower genetic similarity than individuals that share many or rare alleles.

*Step II: Edge pruning (removing edges below a genetic similarity threshold)*

Since in most populations, if considering enough loci, most pairs of individuals will share alleles in at least one locus, the genetic similarity matrix will be very dense and will include very few 0 values, if any. Such dense networks are difficult to analyze, but mainly they include many genetic similarities that are too low to be relevant for analyzing population structure (e.g., two individuals that share a few common alleles are not very informative regarding the existence of subpopulations). Note that “relevant,” in this case, is relative to the population structure resolution we are interested in: if we are interested in a very fine-scale structure, we should focus only on high genetic similarities, while if we are interested in a coarse delineation of structure, we should also incorporate weak genetic similarities in our analysis.

*NetStruct* is designed to analyze population structure at different hierarchical levels, by repeatedly removing edges from the network below genetic similarity thresholds and reapplying the community detection procedure (see *step III*) for each threshold. In the analysis presented in this paper, we first considered the network that consisted of all edges of the constructed genetic similarity network, then the network with all edges below a genetic similarity value of 0.01 removed, then the network with the edges below 0.02 removed, etc. (0 upwards in increments of 0.01). We continued this process, observing the detected communities of each network, until we reached a network in which 10% of the nodes were no longer connected to any other nodes (isolates). In this situation, the genetic similarity threshold is too high to allow meaningful interpretation of the network.

*Step III: Community detection*

For each network (after applying edge-removal), *NetStruct* applies community detection methods according to a specified algorithm. There are currently many community detection methods available (Fortunato, 2010), several of which are supported by *NetStruct*, and all are aimed at identifying dense substructures within networks. The raw outputs of *NetStruct*, at each edge-removal threshold, are the network communities detected by the selected by algorithm (see Fig. S2 for an example).

*Step IV: Testing the null hypothesis*

The *NetStruct* analysis in this paper was aimed at testing the null hypothesis that population structure is not associated with sampling sites. For this purpose, at each edge-removal threshold, the detected communities were arranged as a contingency table according to sampling sites, i.e., the number of individuals assigned to each of the communities for each sampling site. This contingency table for each edge-removal threshold was used for a Fisher’s exact test, with p < 0.05 considered a significant rejection of the null hypothesis. We considered the null hypothesis rejected only if it was rejected for all edge-removal thresholds tested (see Table S3). In this case, rejection of the null hypothesis would suggest that sampling sites are associated with population structure.

**References**

Fortunato, S., 2010. Community detection in graphs. Phys. Rep. 486, 75–174.

Greenbaum, G., Templeton, A.R., Bar-David, S., 2016. Inference and analysis of

population structure using genetic data and network theory. Genetics 202, 1299–1312.
